# Lipoprotein-associated phospholipase A2, myeloperoxidase and vascular endothelial growth factor - predictors of high vascular risk in respiratory bacterial infections

**Published:** 2016

**Authors:** A Seri, DS Marta, A Madalan, M Popescu, AI Tiglea, E Moldoveanu

**Affiliations:** * “Carol Davila” University of Medicine and Pharmacy, Bucharest, Romania; ** “Victor Babes” National Institute of Pathology, Bucharest, Romania; *** “Sf. Pantelimon” Clinical Emergency Hospital, Bucharest, Romania; **** “Titu Maiorescu” University, Faculty of Medicine, Bucharest, Romania

**Keywords:** bacterial respiratory infections, lipoprotein-associated phospholipase A2, myeloperoxidase, vascular endothelial growth factor

## Abstract

**Objective.** Respiratory bacterial infections are associated with important coagulation disturbances that amplify the pulmonary lesions and determine a more severe course of the disease. The aim of our study was to investigate the correlation between the evolution of the general clinical parameters and the occurrence of thrombotic events on one side, and plasma levels of selected proteins involved in inflammation and coagulation on the other side, with the intent to establish and to validate a laboratory test panel for the assessment of the vascular risk in patients with bacterial respiratory infections.

**Methods.** The study included 111 patients (divided into two groups, 61 without thrombosis and 50 with thrombosis) with bacterial respiratory infections and 30 healthy controls, age and gender-matched. The baseline evaluation of the patients included clinical, biological, and respiratory examination.

LpPLA2 and MPO activities were measured by the spectrophotometric method. VEGF was quantified with an ELISA kit.

**Results.** The collected data showed a correlation between the occurrence of superimposed thrombosis in respiratory infection patients, and the intensity of the inflammatory process, reflected by the increased MPO activity, and the dynamics of LpPLA2 and VEGF.

**Conclusion.** Bacterial respiratory infections associate thrombotic vascular events of various degrees of severity, which correlate with the intensity of the inflammatory process, and the severity of endothelium dysfunction at the level of microcirculation. Starting from the recorded data, and based on the established severity scales in use, it is possible to compute a vascular risk score that takes into consideration the values of the three biomarkers under investigation.

**Abbreviations:** COPD = chronic obstructive pulmonary disease,hsCRP = high sensitivity C reactive protein,EC = endothelial cells, ICAM-1 = intercellular adhesion molecule1, LpPLA2 = lipoprotein-associated phospholipase A2, MPO = myeloperoxidase,NK cells = natural killer cells,VEGF = vascular endothelial growth factor, VCAM-1 = vascular cell adhesion molecule 1

## Introduction

Respiratory infections account for an important proportion of the patients admitted in the intensive care unit, either initially, or later, in the course of their illness. Bacterial infections are associated with important coagulation disturbances that amplify the pulmonary inflammatory lesions and determine a more severe course of the disease. Bacterial pathogens and their products trigger the inflammatory response by a transcriptional activation of the inflammatory genes. Simultaneously, anti-inflammatory pathways are also activated, leading to the release of anti-inflammatory cytokines. The exaggerated inflammatory response, compounded by the repression of the anti-inflammatory mechanisms, leads to sepsis [**1**]. The bacterial toxins and other inflammatory mediators released during sepsis generate procoagulant signals that raise the level of the coagulation markers, decrease anticoagulant proteins, and reduce fibrinolytic activity. Numerous cells participate in these processes: endothelial cells, monocytes/ macrophages, neutrophils, thrombocytes, B and T lymphocytes, and NK cells. The sepsis-associated vascular complications negatively affect the patient’s evolution, with thrombotic events sometimes leading to a fatal outcome.

Our study aimed to assess the vascular risk predictive value of clinical parameters, common biological tests, and selected inflammation and coagulation markers in patients admitted in the intensive care unit with respiratory infections, with or without superimposed vascular thrombotic complications.

The clinical parameters (except for those specific to respiratory infections) were focused on acute vascular events, without a long-term follow-up of the endothelial dysfunction initiated by the inflammatory proatherogenic processes. The following elements from the usual laboratory panels were analyzed: blood sugar, hemoglobin, creatinine, CBC with morphology, acute phase reactants (ESR, CRP, fibrinogen), and regular coagulation tests (PT, APTT). 

As a special test to detect procoagulant states, the presence of antiphospholipid antibodies (especially the lupus anticoagulant) was determined in a group of patients, based on the observation that post-infectious antiphospholipid syndrome (sometimes fatal) and low C protein activity occur in severe infections. From the many validated inflammation plasma biomarkers, those concerning procoagulant states were selected too: lipoprotein-associated phospholipase A2 (LpPLA2), myeloperoxidase (MPO), and vascular endothelial growth factor (VEGF).

Having as a rationale the benefits of the preventive coagulation in high-vascular risk patients, we intended to assembly a basic test panel, additionally including selected vascular inflammation biomarkers, which could define the vascular risk profile of the patient with a bacterial respiratory infection.

Lipoprotein-associated phospholipase A2 (LpPLA2) is an enzyme produced in cells involved in atherosclerotic process and is mainly bound to the LDL in the circulation, while the remaining is distributed among HDL, VLDL, and lipoprotein a (Lp(a)). Its activity is correlated with the intensity of atherosclerotic process. LpPLA2 plays a central role in the pathophysiology of atherosclerosis, from its initiation to the development of cardiovascular complications. Numerous epidemiological studies have demonstrated that increased circulating levels of LpPLA2 predict an increased risk of acute cardiovascular events (myocardial infarction, stroke) and cardiovascular mortality [**2**,**3**]. LpPLA2 is a marker of vascular inflammation and represents the link between the lipid metabolism and the low-grade inflammation that is characteristic of cardiovascular or metabolic diseases [**3**]. LpPLA2 has some advantages that make it superior to other inflammatory markers, such as high sensitivity C reactive protein (hsCRP): minimal bio-variation, high specificity for vascular inflammation, and plasma level independent of insulin resistance. The studies performed in last few years have led to the introduction of LpPLA2 in The Cardiovascular Disease Risk Assessment Guidelines in 2008 [**4**], and in The European Guidelines on cardiovascular disease prevention in clinical practice in 2012 [**5**].

Myeloperoxidase (MPO) plays an active role in bacteria phagocytosis and, through its reaction product, HOCl, is directly involved in generating tissue lesions. MPO is released by degranulation of activated leukocytes [**6**].

Endothelial cells (EC) play a pivotal role in thrombosis development in sepsis. Activated EC acquire inflammatory and procoagulant properties. Initially, VEGF was associated with EC survival, proliferation, and migration. Recently, it was established that in addition to its action on EC, VEGF participates to inflammation and coagulation. VEGF induces the expression of cell adhesion molecules [E-selectin, intercellular adhesion molecule1 (ICAM-1) and vascular cell adhesion molecule 1 (VCAM-1)] in EC, and promotes the adhesion of leukocytes. VEGF signaling up-regulates mARN tissue factor, protein and procoagulant activity. VEGF is a late marker of sepsis and there is an association between severe sepsis and elevated circulating level of VEGF. These proinflammatory/ procoagulant effects of VEGF are mediated at least in part, by the activation of NFkB [**7**]. 

## Materials and methods

**Patients.** The study included 111 patients (divided into two groups - 61 without thrombosis and 50 with thrombosis) with bacterial respiratory infections, and 30 healthy controls, age and gender-matched. All the patients were enrolled from patients who presented with a respiratory infection to “Sf. Pantelimon” Clinical Emergency Hospital. The study was approved by the hospital ethics committee and an informed consent was obtained from all the participants. The baseline evaluation of the patients included a clinical, biological, and respiratory examination.

**Inclusion criteria:** patients presenting with bacterial pneumonia (bronchopneumonia), and COPD exacerbations. 

**Exclusion criteria:** malignancy, autoimmune disease, kidney failure, liver cirrhosis, tuberculosis, vascular dementia, Alzheimer disease. 

The patients were diagnosed based on Port, Sofa, and CURB65 algorithms. The microbiological exam was executed from sputum, induced sputum, bronchial aspirate, and hemocultures. The comorbidities present at admission were assessed by a clinical exam, ECG, abdominal and cardiac ultrasound, and radiological exam. The usual laboratory panels obtained were the following: blood sugar, hemoglobin, CBC with morphology, acute phase reactants (ESR, CRP, and fibrinogen), coagulation tests (PT, APTT).

**Inflammation biomarkers.** Plasma obtained from patients and healthy controls was frozen and stored at −80° until analysis. LpPLA2 activity was measured by the spectrophotometric method described by Kosaka et al., by using Azwell Auto LpPLA2 kit and expressed as IU/L [**8**]. The myeloperoxidase activity was determined spectrophotometrically at a wavelength of 405 nm, using o-Dianisidin as substrate [**9**]. Enzymatic activity was expressed in IU/L. VEGF was measured with a commercial ELISA kit (R&D Systems, Germany) and the results were expressed as pg/ml and ng/ml.

**Statistical analysis.** All the values were reported as mean ± standard deviation (SD). Comparisons between groups were carried out by using the analysis of variance (ANOVA). A two tailed p-value<0.05 was considered significant for all the analyses. The statistical analyses were performed with the SPSS software, version 11.0 (SPSS Inc., Chicago, Illinois, USA).

## Results

**Fig. 1:**
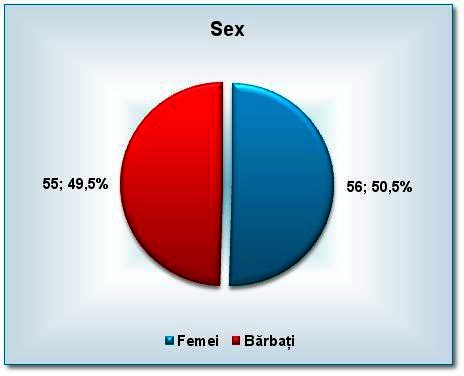
The sex ratio of the patients in the two groups

The sex ratio of the patients in the two groups was balanced (**Fig. 1**).

**Fig. 2:**
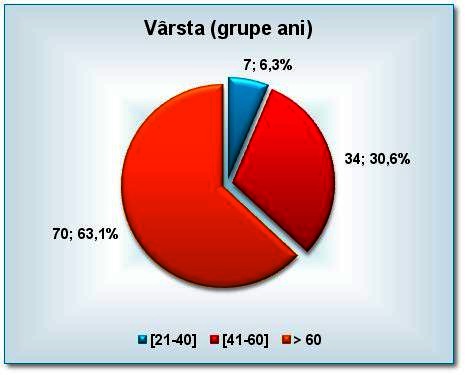
The age distribution of the patients

The age distribution of the patients showed a predominance of patients over 60 years in both groups, without any statistical significant difference between the two groups of patients. What should be noted is that age can be a risk factor (**Fig. 2**).

**Fig. 3:**
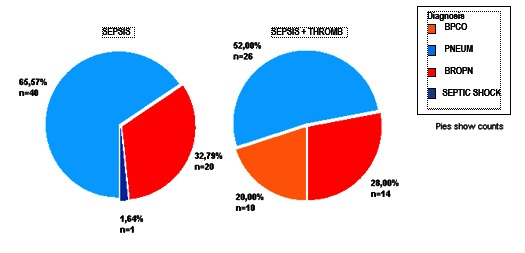
The distribution of patients according to the diagnosis

In both groups, pneumonia was the most prevalent nosological entity (**Fig. 3**).

**Fig. 4:**
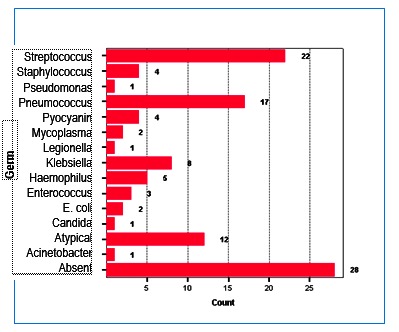
Distribution of etiological agents

The infectious agent was isolated in 83 patients; the most prevalent were Streptococcus sp., Pneumococcus, and Chlamydia pneumoniae (included in the figure under the heading “atypical”) (**Fig. 4**).

Among the thrombotic events that compounded the clinical course of the patients, the most prevalent were stroke (24%), pulmonary thromboembolism of various degrees of severity (16%), and acute coronary syndromes (acute myocardial infarction and unstable angina, with a cumulated incidence of 30%).

**Fig. 5:**
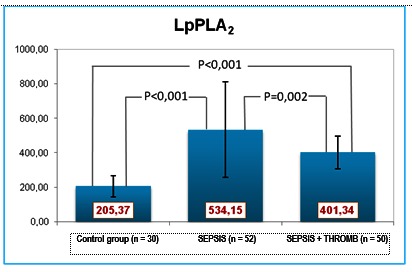
LpPA2 values

LpPLA2 showed statistically significant elevations in both groups vs. controls, with the highest values in group 1 (**Fig. 5**).

**Fig. 6:**
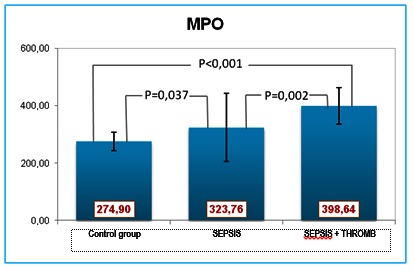
MPO values

MPO showed statistically significant elevations in all patients compared with the controls, with higher values in the second group (**Fig. 6**).

**Fig. 7:**
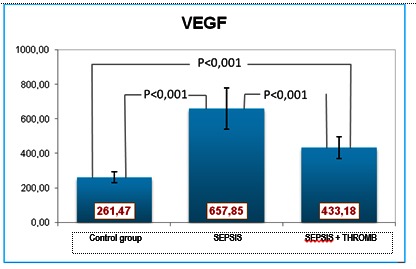
VEGF values

VEGF was elevated in all patients, more prominently in the first group (**Fig. 7**).

**Fig. 8:**
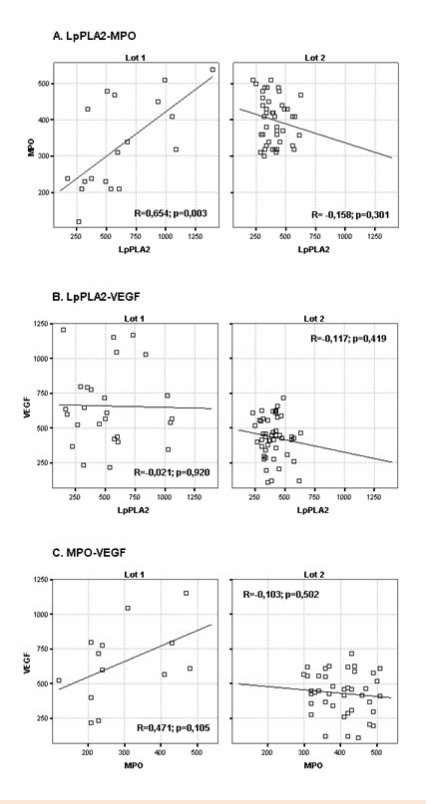
The correlations between the studied biomarkers. A. LpPLA2- MPO/ B. LpPLA2-VEGF/ C. MPO-VEGF

The Pearson correlation coefficient (R) was calculated between all the pairs of the investigated biomarkers (**Fig. 8**).

## Discussion

Respiratory infections have a wide range of clinical expressions, from resolving infection to sepsis, and to septic shock. They induce an inflammatory syndrome triggered by the interaction of the host with the microbial pathogens, a syndrome frequently associated with microvascular endothelial dysfunction [**10**]. The severity of the patient’s response to the bacterial aggression depends on the type of the bacteria involved in the infective process, the polymicrobial nature of the infection, and the host’s reactivity regarding the inflammatory response and the development of a prothrombotic state. The subjects showed differences in the hospital stay length depending on the germ type.

The myeloperoxidase released by the activated cells acts upon the platelets, activating them and, promoting their aggregation. Additionally, the myeloperoxidase associates with integrins and activates the polymorphonuclear neutrophil leukocytes, leading to adhesion molecules exposure and, ultimately, to the binding of fibrinogen (the main glycoprotein IIb/IIIa ligand on the platelet surface) to the serum and the formation of a stable platelet aggregate. This is one of the intravascular clot-generating pathways and a bridge between inflammation and coagulation.

The positive MPO-LpPLA2 correlation within group I, and the negative correlation within group II, was likely caused by the more severe endothelial dysfunction of the patients in group II. Within group 1, LpPLA2 also displayeda positive correlation with VEGF. The pulmonary infection and the inflammatory environment (especially within the epithelium of the airways) can up-regulate the production of LpPLA2, which is the main source of lysophosphatidylcholine present in increased amounts in serum and bronchial secretions. The severity of the infection/inflammation in the second group of patients led to the expectation of higher LpPLA2 values, which was not substantiated eventually. Contrary to the expectations, the LpPLA2 levels were low, and associated low VEGF and high MPO. In severe pulmonary disease, VGF levels decline as a result of the endothelial dysfunction and exhaustion of the repair mechanisms [**11**,**12**].

Three major anticoagulant pathways regulate the activation of coagulation: antithrombin, protein C system, and tissue factor pathways.The function of all the three pathways is impaired during the inflammation-induced activation of the coagulation [**13**]. 

A scale-up of the study, whichwould include a larger patient population, would yield data that are more conclusive.

## Conclusions

Bacterial respiratory infections associate thrombotic vascular events of various degrees of severity, which correlate with the intensity of the inflammatory process, and the severity of endothelium dysfunction at the level of microcirculation. Based on the presented data, and in accordance with the standardized severity scores already in use, it is possible to compute a vascular risk score for the patient with respiratory infections, based in part on the values of the three studied biomarkers.
